# Fatal presentation of congenital neuroblastoma with placental metastases: report of a rare case

**DOI:** 10.1007/s00247-026-06684-1

**Published:** 2026-06-06

**Authors:** Mohammad Obeidat, Ahmad Huneity, Yaman M. Alahmad, Lina Irshaid, Sara Alziyoud, Hanan Alobaidli, Amal Al-Rashid

**Affiliations:** 1 Hamad Medical Corporation, 3050, Doha, Qatar; 2 Qatar University, Doha, Qatar; 3 Doha, Qatar

**Keywords:** Adrenal gland neoplasms, Fetal death, intrauterine, Fetal pathology, Neuroblastoma, congenital, Placental neoplasms, Prenatal ultrasonography

## Abstract

Congenital neuroblastoma is the most common malignant fetal tumor, yet placental metastases are exceptionally rare. We report a fetus with a right-sided suprarenal mass, hydrops, and marked placentomegaly that progressed to intrauterine demise at 26 weeks. Histopathology confirmed widespread metastatic neuroblastoma involving fetal organs and placental fetal vessels while sparing maternal intervillous spaces. This case is unique due to extensive involvement of placental fetal vessels with complete sparing of the maternal intervillous space. It highlights the importance of considering metastatic neuroblastoma when fetal abdominal masses coexist with hydrops and placentomegaly.

**Supplementary Information:**

The online version contains supplementary material available at 10.1007/s00247-026-06684-1.

## Introduction

Malignant fetal tumors are uncommon, and placental involvement in such cases is even rarer [1]. Among these, neuroblastoma is the most frequent fetal malignancy, accounting for one-third of congenital cancers with an estimated incidence ranging from 1 in 10,000 to 30,000 live births [2, 3]. To date, fewer than 20 cases of placental involvement have been reported in the English-language literature [1]. Fetal neuroblastoma usually originates in the adrenal gland, often presenting as a solitary suprarenal mass [4]. Placental metastases from fetal neuroblastoma are exceedingly rare yet represent the most common malignancy associated with fetal-to-placental spread [5].

## Case presentation

Informed consent was obtained from patient/parent for publishing the case report.

We report a case of a 26-year-old pregnant woman, gravida 2 para 1, with no significant medical, obstetric, gynecological, surgical, or family history, who presented at 26 weeks and 3 days of gestation with lower abdominal pain and decreased fetal movement, without vaginal bleeding.

Her vital signs and physical examination were unremarkable, and bedside Doppler ultrasonography confirmed fetal cardiac activity. Initial basic laboratory tests including complete blood count and renal and liver function tests were within normal limits.

**Fig. 1:**
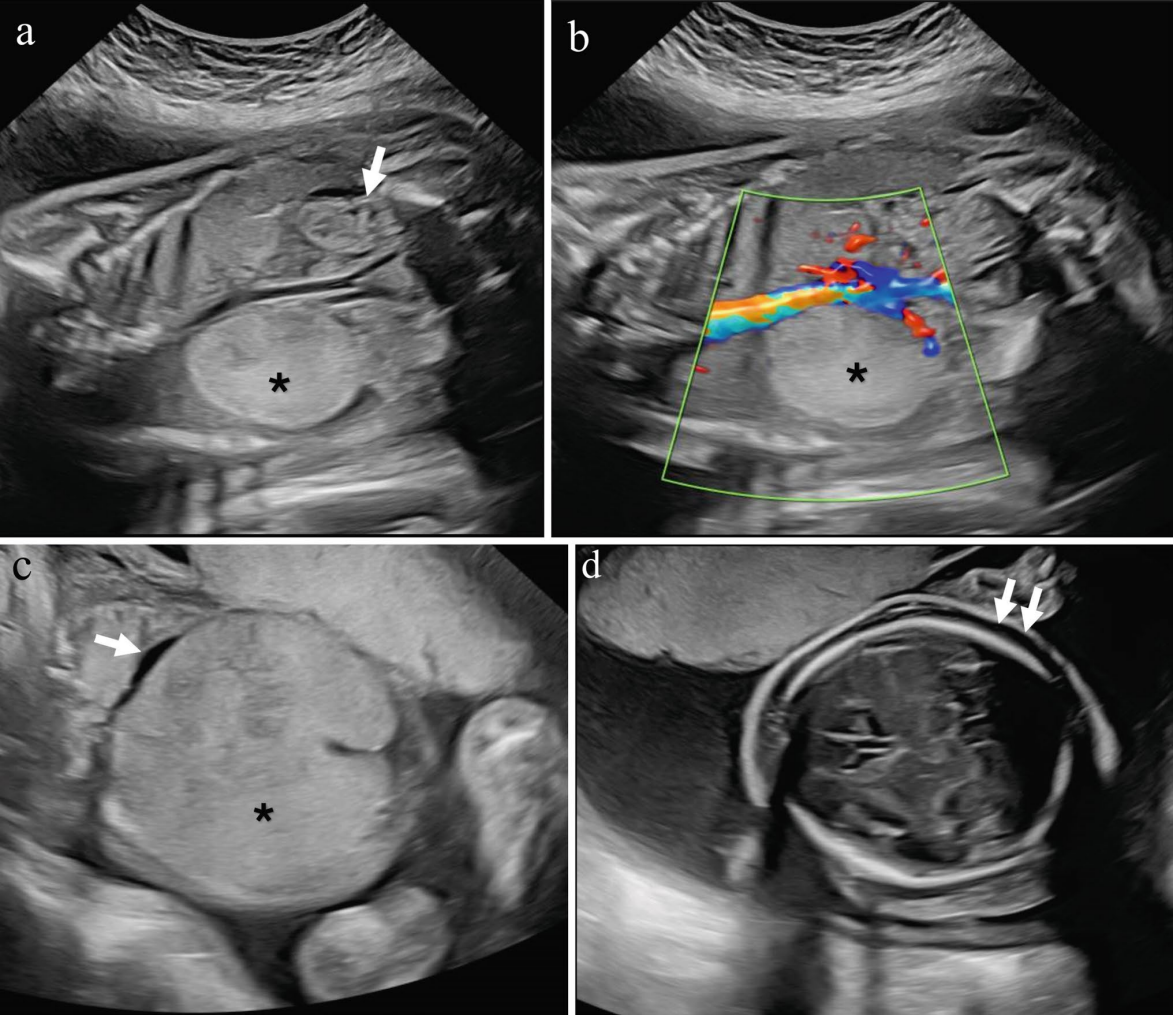
Ultrasound images of a 26-week gestational age male fetus with congenital neuroblastoma and placental metastases. **a** Grayscale longitudinal ultrasound image of the fetal abdomen reveals a well-defined, oval-shaped homogeneous hyperechoic lesion (*asterisk*) in the right suprarenal region, inseparable from the right kidney. The left kidney appears normal (*arrow*). **b** Longitudinal doppler image of the lesion (*asterisk*) shows no appreciable internal vascularity. **c** Grayscale longitudinal image of the fetal liver demonstrates hepatomegaly with mild heterogeneous echotexture and no focal lesions (*asterisk*), and mild ascites (*arrow*). **d** Grayscale transverse image of the fetal brain demonstrates diffuse scalp edema (*arrows*)

**Fig. 2:**
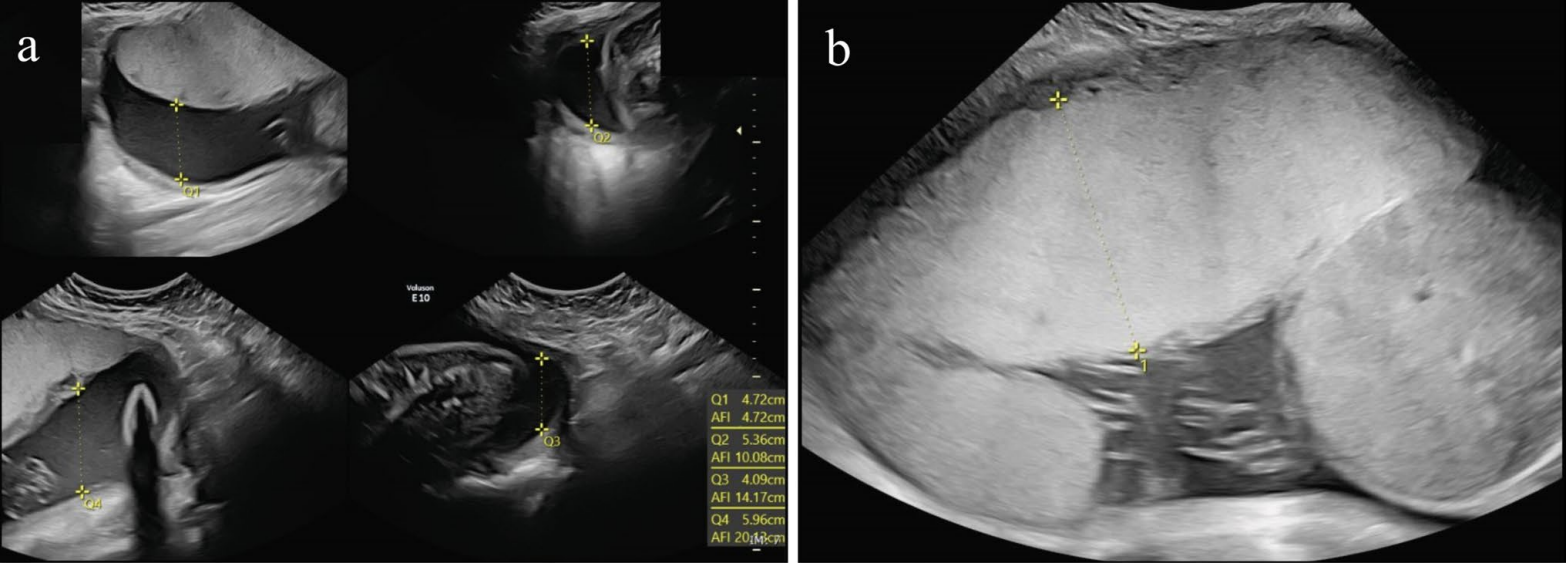
Ultrasound images of a 26-week gestational age male fetus with congenital neuroblastoma and placental metastases. **a** Grayscale ultrasound images demonstrate the four-quadrant amniotic fluid index (AFI) measuring 20 cm (upper limit of normal to mild polyhydramnios). **b** Grayscale ultrasound image shows bulky, thickened, and slightly heterogeneous placenta measuring 7 cm in thickness with no appreciable focal lesions. *AFI* amniotic fluid index

Emergency obstetric ultrasound revealed a viable singleton fetus with a well-defined oval-shaped, homogeneous hyperechoic mass in the right suprarenal region, inseparable from the right kidney. It also showed features of hydrops fetalis, including fetal hepatomegaly, diffuse scalp edema, and mild ascites (Fig. 1). Placentomegaly and upper limit of normal to mild polyhydramnios were identified (Fig. 2). Fetal head and abdominal growth parameters exceeded the 99th percentile, likely reflecting edema-related overestimation secondary to hydrops rather than true fetal macrosomia. Doppler ultrasound of the umbilical and middle cerebral arteries showed normal waveforms and indices.

One week later, upon referral to the fetal-maternal unit, a follow-up ultrasonography demonstrated absent fetal cardiac activity. A shared decision between the treating team and the patient was made to terminate the pregnancy, and an emergent cesarean section was performed.

**Fig. 3:**
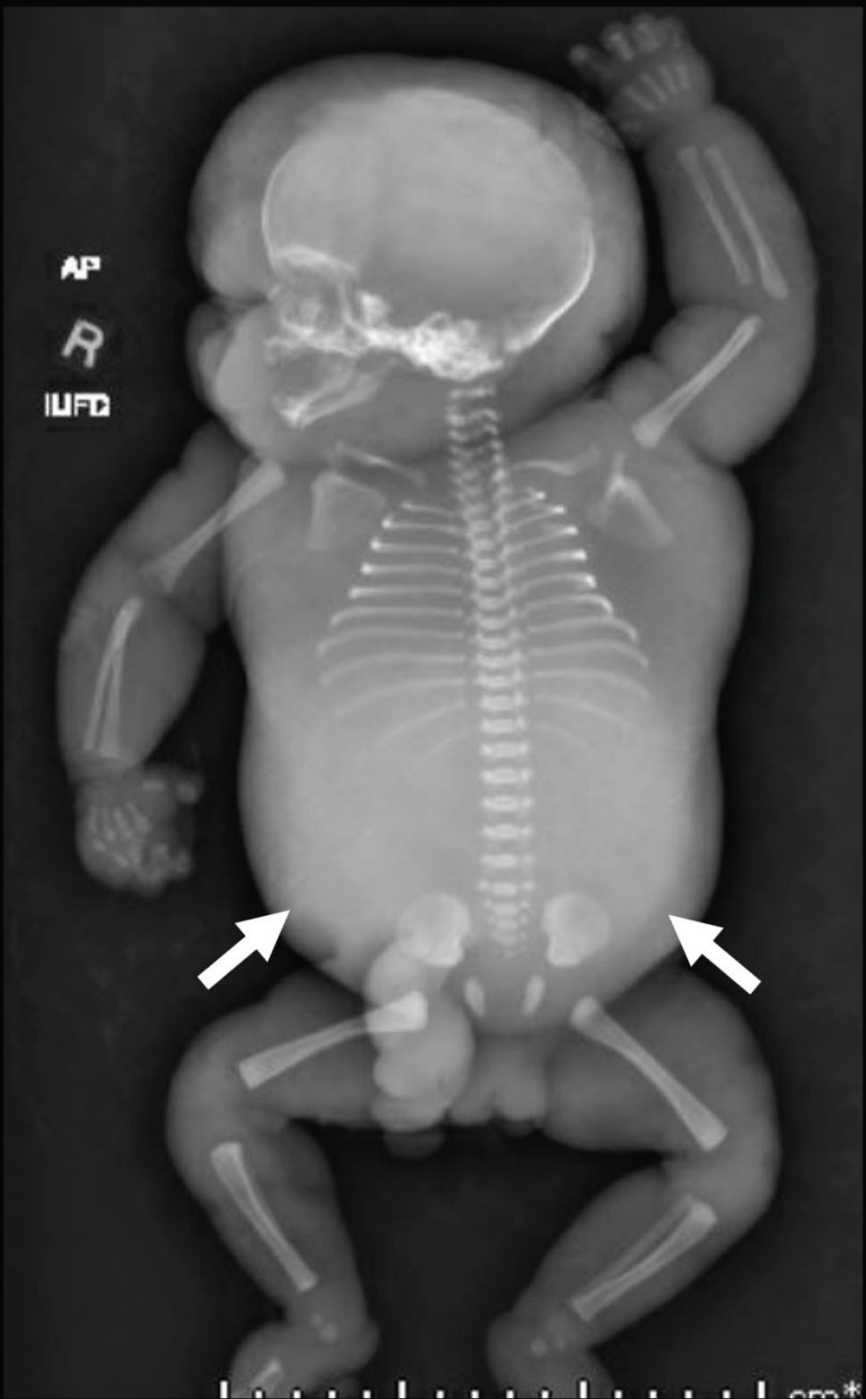
Postmortem radiograph of a 26-week gestational age fetus with congenital neuroblastoma and placental metastases. Anteroposterior radiograph (babygram) demonstrates generalized soft tissue edema and abdominal distension (*arrows*). No skeletal abnormalities are identified

A postmortem anteroposterior babygram (radiograph) showed marked soft tissue edema and abdominal distension without bony lesions (Fig. 3).

The patient subsequently developed postpartum hypertension without end-organ damage, which was managed with antihypertensive therapy. Her blood pressure returned to normal baseline, and she was discharged home 5 days later.

**Fig. 4:**
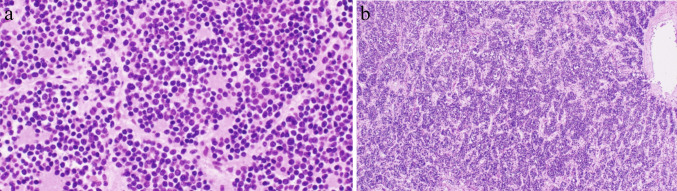
Histopathological images of a 26-week gestational age fetus with congenital neuroblastoma and placental metastases. **a** Histopathological image of the fetal suprarenal mass (×40) shows sheet-like growth of small round blue cells; these cells display high nuclear-to-cytoplasmic ratios, salt-and-pepper chromatin, and multiple Homer-Wright pseudorosettes. **b** Histopathological image of the fetal liver (×4) shows similar small round blue cells

Autopsy of the fetus demonstrated generalized edema, ascites, right nephromegaly, and hepatomegaly occupying most of the abdomen. Histology sections of the kidney and liver (Fig. 4) showed extensive infiltration by small round blue cells, sharing the same morphology and immunophenotype as the placental tumor indicating neuroblastoma. Heart capillary vessels showed similar tumor cells.

On gross examination, there was a singleton discoid placenta, measuring 22×20×3.5 cm and weighing 1,400 g (without membranes and cord), exceeding the 95th percentile for gestational age. The membranes appeared dusky-gray and were marginally inserted. The three-vessel umbilical cord was normally coiled, inserted pericentrally, and had a smooth, white, glistening external surface. Both the maternal and fetal surfaces appeared unremarkable. Upon serial sectioning, the placental parenchyma was tan-pink, firm, and homogeneous.

**Fig. 5:**
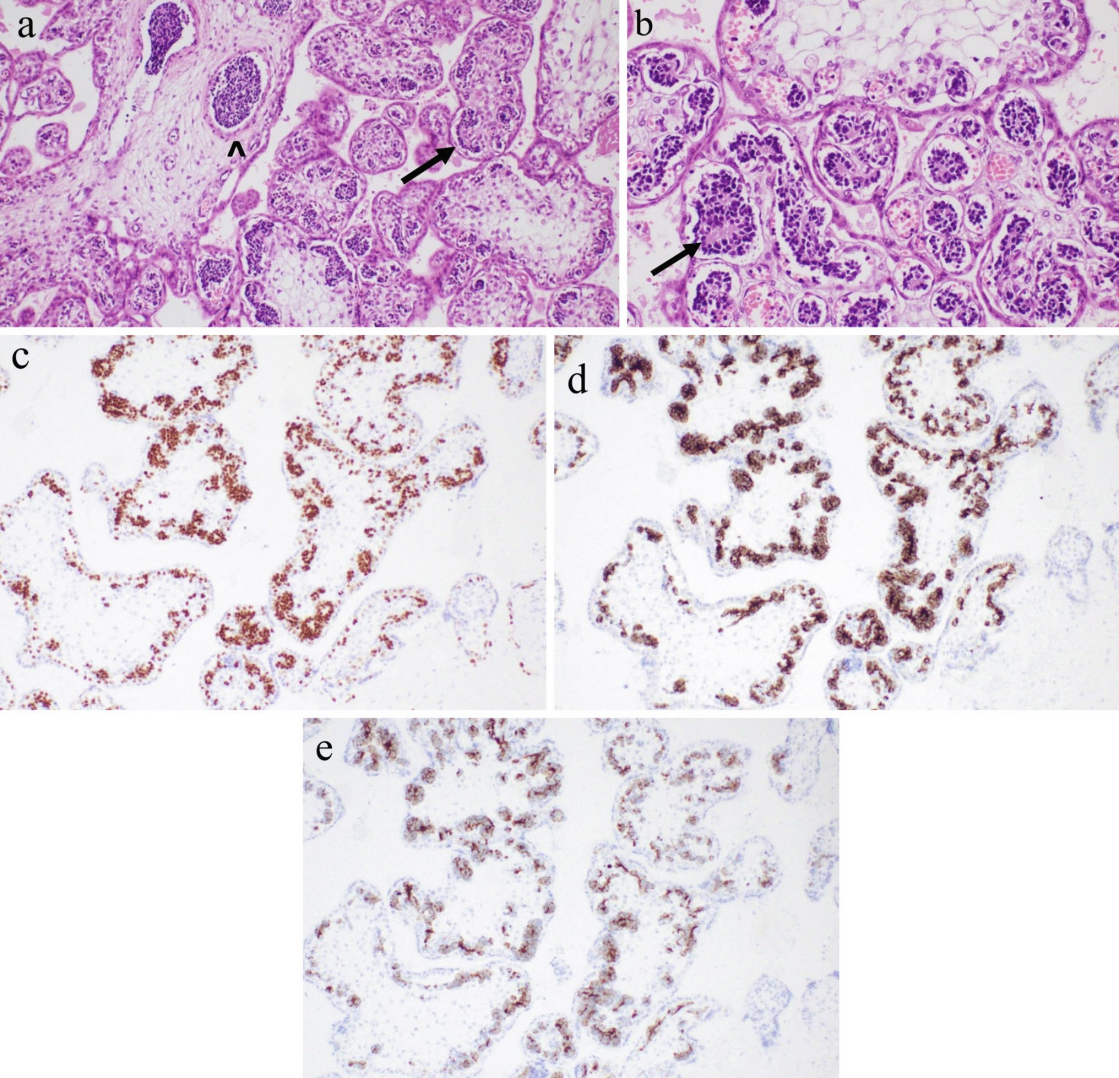
Microscopic and immunohistochemical findings of the placenta in a 26-week gestational age fetus with congenital neuroblastoma and placental metastases. **a** Microscopic examination of the placenta on low-power view (×10) confirms diffuse involvement of the fetal vascular tree by neuroblastoma; no evidence of maternal vascular involvement is identified. Both chorionic villous vessels (*arrow*) and more proximal stem villous vessels (*arrowhead*) are involved. **b** Microscopic examination of the placenta on a higher magnification view (×20) shows involvement of both chorionic villous vessels (*arrow*). **c** Immunohistochemical GATA3 stain image (×10) shows positive tumor cell staining. **d** Immunohistochemical synaptophysin stain image (×10) shows positive tumor cell staining. **e** Immunohistochemical chromogranin A stain image (×10) shows positive tumor cell staining

Microscopically (Fig. 5), the placental parenchyma demonstrated maturation consistent with gestational age. At low power, diffuse involvement of chorionic and stem villous vessels by a population of discohesive small round blue cells was noted. At higher magnification, these cells exhibited high nuclear-to-cytoplasmic ratios, salt-and-pepper chromatin, and Homer-Wright pseudorosettes surrounding eosinophilic neuropil. Similar neoplastic cells were identified within the umbilical veins. No tumor involvement was seen in the intervillous space, and the membranes appeared unremarkable. Immunohistochemistry (with appropriate controls) demonstrated the tumor cells were positive for GATA3 (a marker reported in neuroblastoma and supporting neural crest differentiation), synaptophysin, and chromogranin A, and had an elevated Ki-67 proliferation index. Tumor cells were negative for EMA, CK AE1/AE3, MNF116, S100, NKX2.2, CD45, CD20, CD3, CD99, WT-1, Myo-D1, and desmin. The overall morphologic and immunophenotypic features were consistent with neuroblastoma.

## Discussion

Neuroblastoma originates from primordial neural crest cells that constitute the sympathetic ganglia [6]. Most fetal neuroblastomas are diagnosed in the third trimester; however, earlier detection has been reported as early as 19 weeks to 20 weeks of gestation [4].

This report describes a case of fetal neuroblastoma with widespread fetal and placental metastases which was diagnosed at 26 weeks’ gestation.

Neuroblastomas are often located suprarenally and on the right side. About 50% of these tumors are cystic, albeit they may present as solid or complicated masses on ultrasound [7]. They usually appear as well-encapsulated suprarenal masses that displace the kidney inferolaterally [7, 8].

The neuroblastoma in our case appeared as a solid hyperechoic mass which was inseparable from the right kidney, suggesting renal involvement, as confirmed by histopathology. Key differential diagnoses for this imaging finding include adrenal hemorrhage, subdiaphragmatic masses, and renal neoplasms.

The most common site of metastasis for neuroblastoma is the liver, accounting for 25% of the cases, which can be diffuse or mass-like patterns [2, 6, 8]. Placental involvement has also been reported [1]. Although the ultrasound in our case did not show discrete focal hepatic or placental masses, the findings of hepatomegaly and increased placental thickness suggested infiltration. The development of fetal hydrops has been discussed in previous studies and can be possibly explained by the excess of catecholamines, liver involvement, and erythropoietic tissue invasion [8].

Similar to our case, the placental metastases were confined to chorionic and stem villous vessels with sparing of the maternal intervillous space, possibly related to suppression of the fetal tumor cells by the maternal immune system [1]. To the best of our knowledge, there was a single case by Kume et al. that reported involvement of the maternal intervillous space of the placenta [1]. It is noteworthy that in many regions, including ours, postmortem examinations are often culturally sensitive and may face limited acceptance.

Finally, to the best of our knowledge, there are a total of 13 reported cases of congenital neuroblastoma with placental metastases (Supplementary Material 1).

## Conclusion

Fetal neuroblastoma is the most common malignant fetal tumor with potential for placental metastases. Early detection by fetal ultrasound is crucial for the diagnosis and management. Metastatic neuroblastoma should always be considered in patients presenting with fetal intra-abdominal mass, increased placental thickness, and features of hydrops. Definitive diagnosis remains dependent on meticulous autopsy and histopathological and immunohistochemical examination.

## Supplementary Information

Below is the link to the electronic supplementary material.

ESM 1 (DOCX.17.0 KB)

## Data Availability

No datasets were generated or analysed during the current study.

## References

[1] Kume A, Morikawa T, Ogawa M, Yamashita A, Yamaguchi S, Fukayama M (2014) Congenital neuroblastoma with placental involvement. Int J Clin Exp Pathol 7(11):8198–204 PMC4270547 25550872

[2] Acharya S, Jayabose S, Kogan SJ, et al (1997) Prenatally diagnosed neuroblastoma. Cancer 80:304–310 9217044 10.1002/(sici)1097-0142(19970715)80:2<304::aid-cncr19>3.0.co;2-y

[3] Heling KS, Chaoui R, Hartung J, et al (1999) Prenatal diagnosis of congenital neuroblastoma. Fetal Diagn Ther 14:47–52 10072651 10.1159/000020888

[4] Miric Tesanic D, Habek D, Vasilj O, Stanojevic M (2010) Metastatic fetal neuroblastoma with non immune fetal hydrops. Ultraschall Med 31:520–522 20091466 10.1055/s-0028-1109870

[5] Roberts DJ, Oliva E (2006) Clinical significance of placental examination in perinatal medicine. J Matern Fetal Neonatal Med 19:255–264 16753764 10.1080/14767050600676349

[6] Lonergan GJ, Schwab CM, Suarez ES, Carlson CL (2002) Neuroblastoma, ganglioneuroblastoma, and ganglioneuroma: radiologic–pathologic correlation. Radiographics 22:911–934 12110723 10.1148/radiographics.22.4.g02jl15911

[7] Kesrouani A, Duchatel F, Seilanian M, Muray JM (1999) Prenatal diagnosis of adrenal neuroblastoma by ultrasound: a report of two cases and review of the literature. Ultrasound Obstet Gynecol 13:446–449 10423810 10.1046/j.1469-0705.1999.13060446.x

[8] Allen AT, Dress AF, Moore WF (2007) Mirror syndrome resulting from metastatic congenital neuroblastoma. Int J Gynecol Pathol 26:310–312 17581417 10.1097/pgp.0b013e31802e3bfe

