# Supplementary material for: Fatal presentation of congenital neuroblastoma with placental metastases: report of a rare case
Source: Pediatr Radiol. 2026 Jun 6;56(8):1814–9. doi: 10.1007/s00247-026-06684-1 (PMC13407455; doi:10.1007/s00247-026-06684-1)
Supplement: Supplementary file 1 — (DOCX.17.0 KB) [file 247_2026_6684_MOESM1_ESM.docx]

Table 1: Reported cases of placental metastasis from congenital neuroblastoma.

| Year | Author(s) | Cases | Key Finding | Reference |
| --- | --- | --- | --- | --- |
| 1964 | Strauss & Driscoll | 2 | First description of congenital neuroblastoma involving placenta | Congenital neuroblastoma involving the placenta. reports of two cases. Pediatrics. PMID: 14181981. |
| 1973 | Anders et al. | 2 | Metastasizing fetal neuroblastoma involving placenta | Metastasizing fetal neuroblastoma with involvement of the placenta simulating fetal erythroblastosis: Report of two cases. https://doi.org/10.1016/S0022-3476(73)80009-5. |
| 1981 | Smith, Chan & deSa | 2 | Placental tumor emboli confined to fetal circulation | “Placental involvement in congenital neuroblastoma.” Journal of Clinical Pathology 34: 785-789. |
| 1997 | Lynn et al. | 1 | Disseminated congenital neuroblastoma with placental metastasis | “Disseminated congenital neuroblastoma involving the placenta.” Archives of pathology & laboratory medicine 121 7: 741-4 . |
| 1999 | Ohyama et al. | 1 | Placental pathology helped establish diagnosis | Congenital neuroblastoma diagnosed by placental examination. Medical and Pediatric Oncology. https://doi.org/10.1002/(SICI)1096-911X(199910)33:4<430::AID-MPO23>3.0.CO;2-P |
| 2007 | Allen et al. | 1 | Placental metastasis associated with mirror syndrome | Mirror syndrome resulting from metastatic congenital neuroblastoma. International Journal of Gynecological Pathology. PMID: 17581417. |
| 2010 | Miric-Tesanic et al. | 1 | Metastatic fetal neuroblastoma with hydrops | Metastatic fetal neuroblastoma with non immune fetal hydrops. Ultraschall in der Medizin. PMID: 20091466. |
| 2014 | Kume et al. | 1 | Tumor cells in chorionic villi and intervillous space | Congenital neuroblastoma with placental involvement. International Journal of Clinical and Experimental Pathology. PMID: 25550872. |
| 2018 | Korean case (mirror syndrome) | 1 | Placental metastatic neuroblastoma causing maternal complications | Mirror Syndrome Resulting from Metastatic Congenital Neuroblastoma to Placenta. Perinatology. https://doi.org/10.14734/PN.2018.29.4.189 |
| 2024 | Campillo-Ajenjo et al. | 1 | Congenital metastatic neuroblastoma with placental involvement | Congenital metastatic neuroblastoma with placental involvement as a rare cause of non-immune fetal hydrops. Journal of Obstetrics and Gynaecology Research. https://doi.org/10.1111/jog.15968 |
